# Supplementary material for: Conditional internalization of PEGylated nanomedicines by PEG engagers for triple negative breast cancer therapy
Source: Nat Commun. 2017 Jun 8;8:15507. doi: 10.1038/ncomms15507 (PMC5472176; doi:10.1038/ncomms15507)
Supplement: Supplementary Information — Supplementary Figures, Supplementary Methods and Supplementary References [file ncomms15507-s1.pdf]

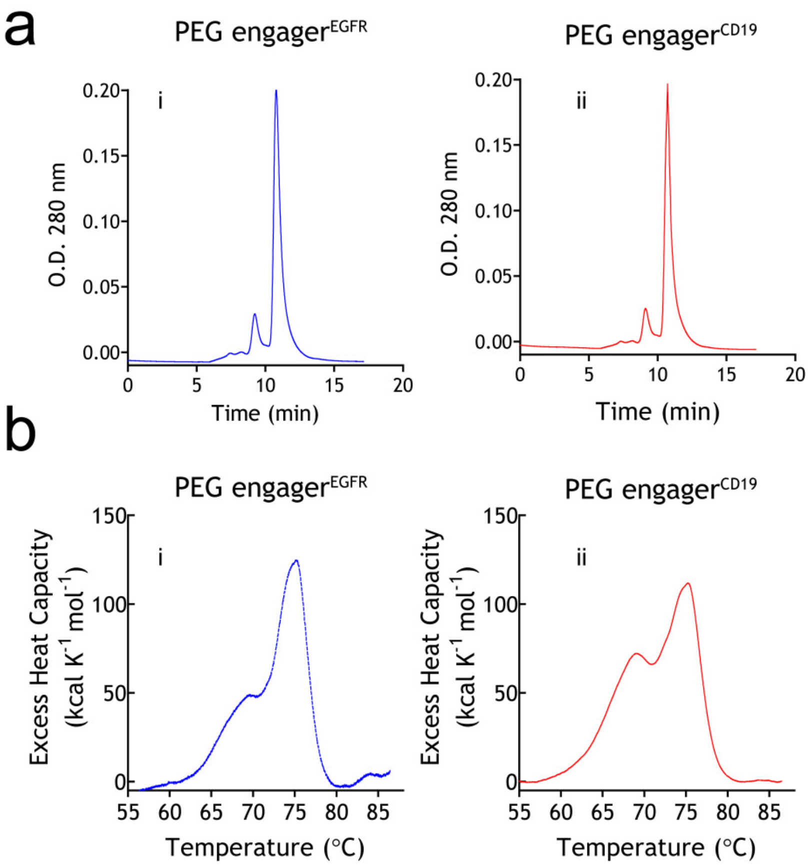

Supplementary Figure 1. Characterization of recombinant PEG engagers. (a) Size-exclusion high-performance liquid chromatography of PEG engager^{EGFR} (i) and PEG engager^{CD19} (ii). (b) Thermal unfolding of PEG engager^{EGFR} (i) and PEG engager^{CD19} (ii) as measured by differential scanning calorimetry in PBS at a heating rate of 1°C per min.

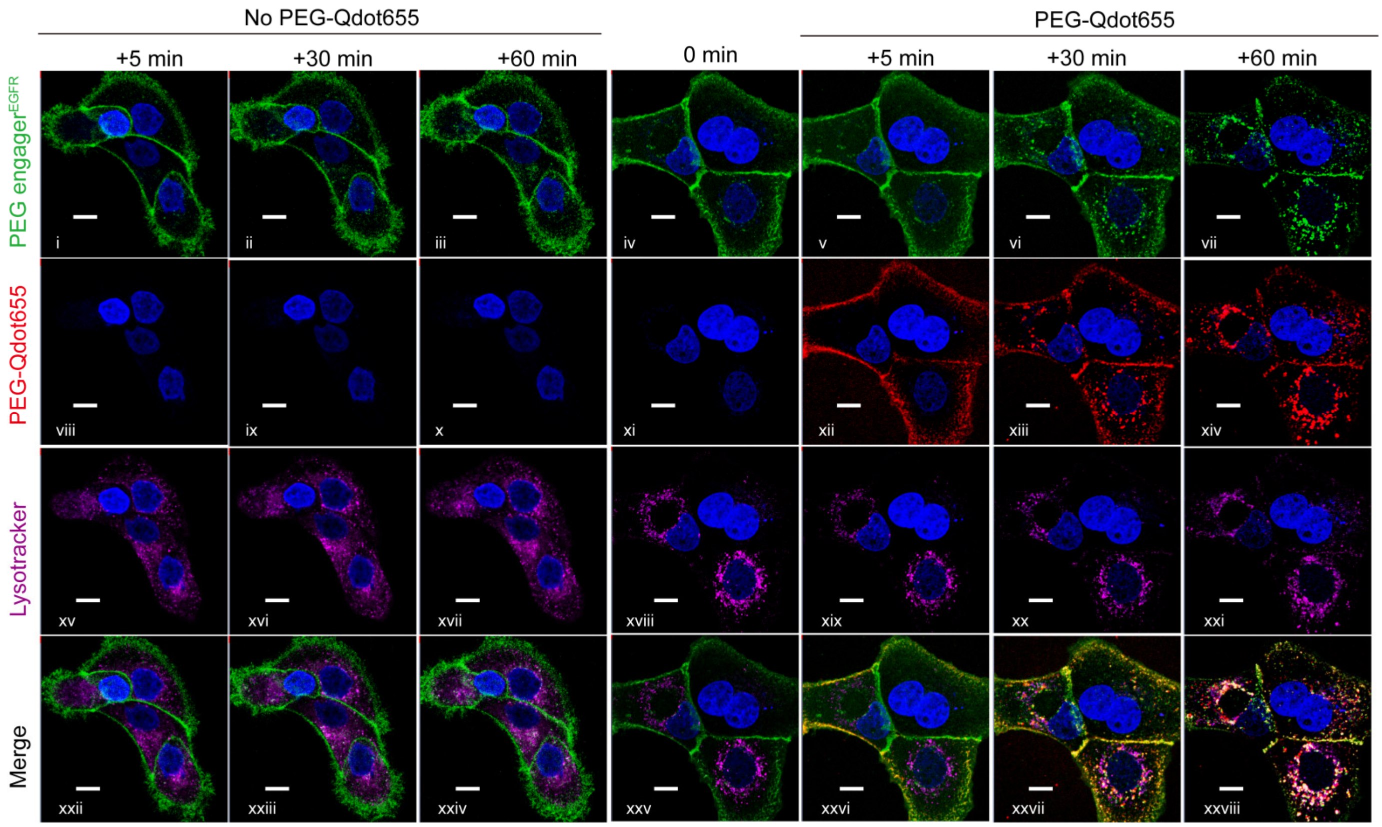

Supplementary Figure 2. Conditional internalization of PEGylated nanoparticles in BT20 cells. Fluorescent-labeled PEG engager^{EGFR} on the cell membrane of BT20 cells after 1 h at 37°C (green, iv) was real-time imaged 5 min, 30 min and 60 min after incubation without (i-iii) or with (v-vii) PEG-Qdot655. Hoechst 33342 (blue), PEG-Qdot655 (red, viii-xiv) and LysoTracker Red DND-99 (purple pseudo color, xv-xxi) indicate nucleus, Qdot and lysosome staining, respectively. Merged images are shown in panels xxii-xxviii. Scale bars, 10 µm.

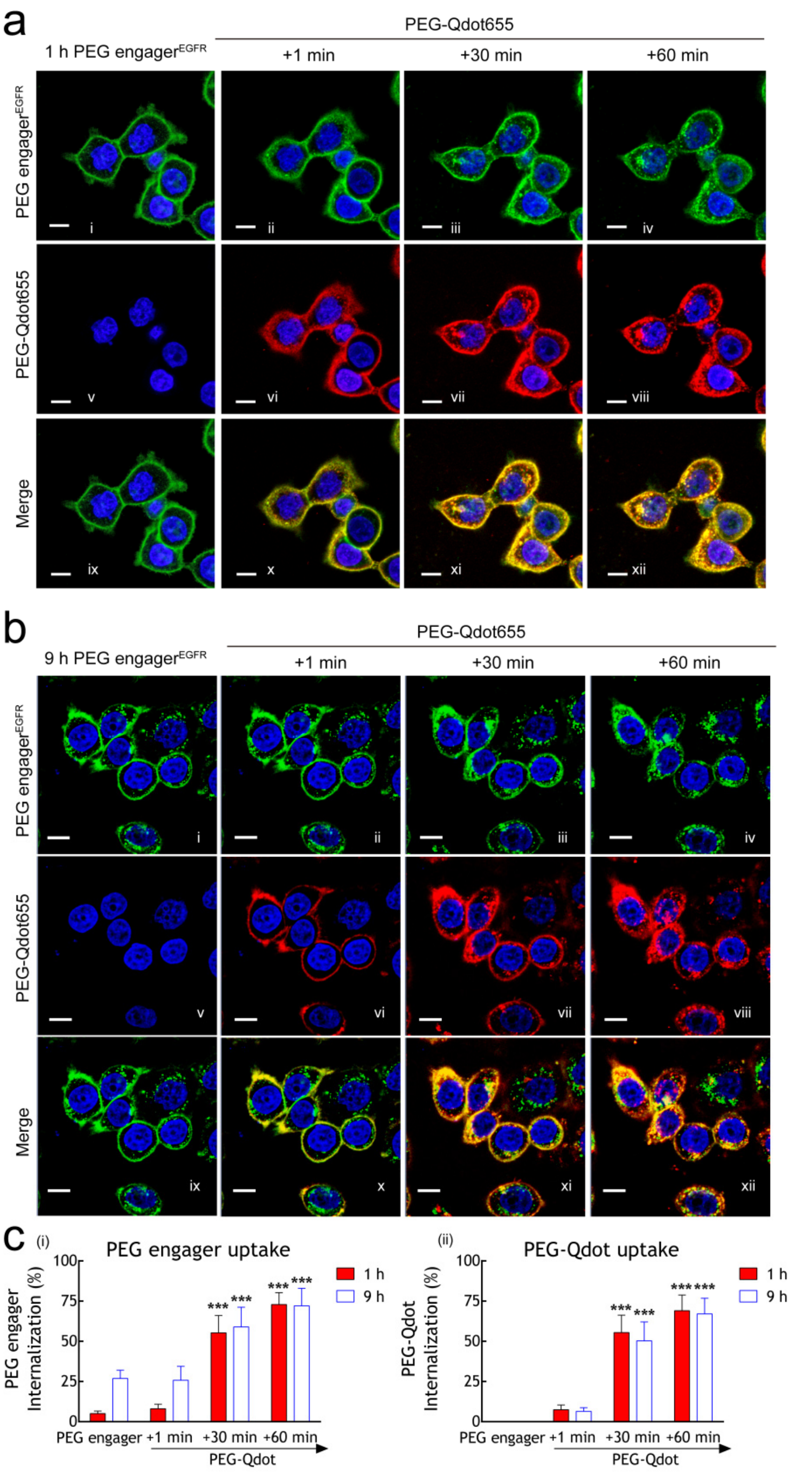

Supplementary Figure 3. Conditional internalization of PEGylated nanoparticles by PEG engagers. Pre-targeted fluorescent-labeled PEG engager^{EGFR} (green) on the cell membrane of MDA-MB-468 cells was real-time imaged 1 h (a, i) or 9 h (b, i) after antibody addition. PEG engager^{EGFR} (green) and PEG-QDot655 (red, v-viii) were imaged 1 min, 30 min and 60 min after addition of PEG-Qdot655. Hoechst 33342 (blue) for nucleus staining. Merged images are shown in panels ix-xii. Scale bars, 10 μm. (c) Percentage of the PEG engager^{EGFR} (i) or PEG-Qdot655 (ii) that internalized into cells at different times was quantified from confocal images of individual cells (n=16). Representative confocal images from two independent experiments are shown. Data are shown as mean ± standard deviation. Significant differences in percentage of internalization before and after addition of PEG-Qdot655 are indicated: ***, $p \leq 0.0001$ (two-way analysis of variance).

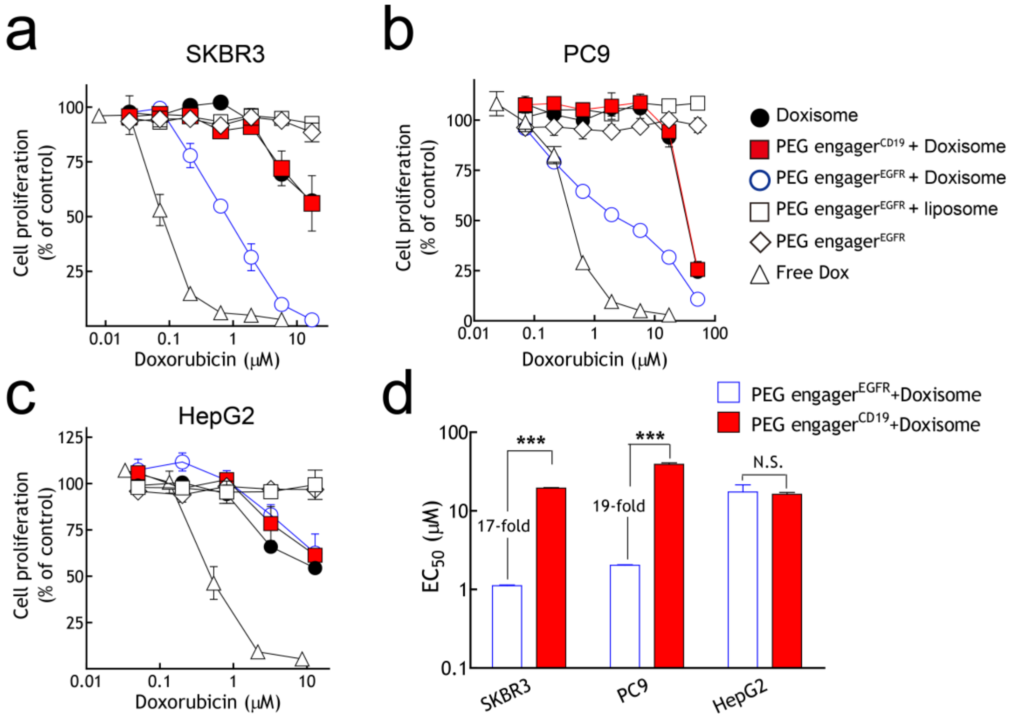

Supplementary Figure 4. PEG engager enhances the anti-proliferative activity of Doxisome®. SKBR3 (a), PC9 (b), and HepG2 cells (c) were incubated with PEG engager^{EGFR} (white circles), PEG engager^{CD19} (red squares) or culture medium (black circles) for 30 min before addition of serial dilutions of free Doxisome® (liposomal doxorubicin) in triplicate for 4 h. Serial dilutions of doxorubicin (white triangles), PEG engager^{EGFR} (white diamonds) or PEG engager^{EGFR} followed by serial dilutions of empty liposomes (white squares) were also added to cells in triplicate for 4 h. The incorporation of ^{3}H-thymidine into cellular DNA was measured 72 h later. The data are representative of three independent experiments. (d) The half maximal effective concentration (EC_{50}) values of SKBR3, PC9 and HepG2 cells treated with PEG engager^{CD19} plus Doxisome® or PEG engager^{EGFR} plus Doxisome® were analyzed (n = 3). Data are shown as mean ± standard deviation. Significant differences in mean EC_{50} values are indicated: ***, $p \leq 0.0001$ (two-way analysis of variance). N.S., not significant.

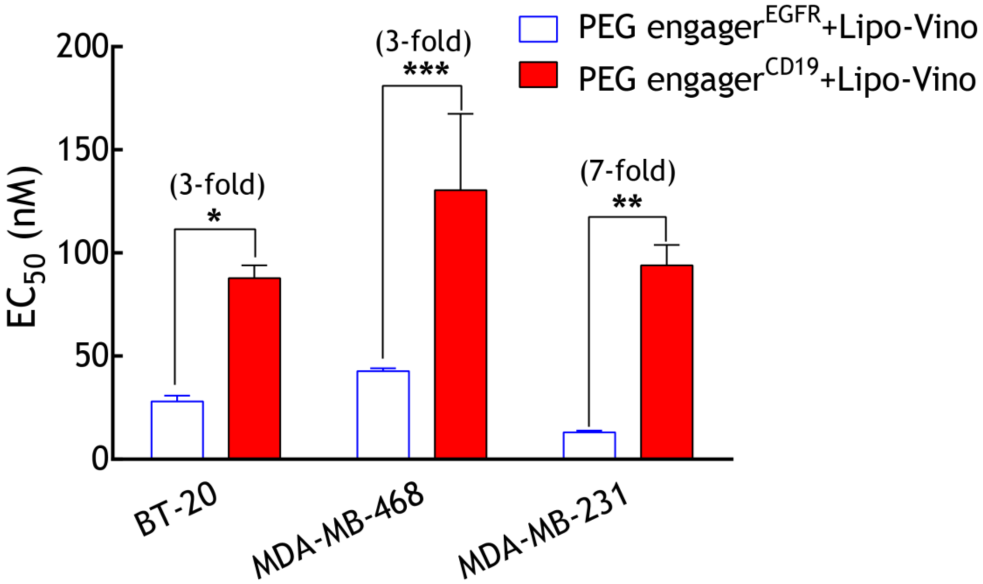

Supplementary Figure 5. PEG engager enhances the anti-proliferative activity of liposomal vinorelbine. BT-20, MDA-MB-468, and MDA-MB-231 cells were incubated with PEG engager^{EGFR} (white bars) or PEG engager^{CD19} (red bars) for 30 min followed by serial dilutions of liposomal vinorelbine (Lipo-Vino) in triplicate for 4 h. The incorporation of ^{3}H-thymidine into cellular DNA was measured 72 h later. The data are representative of three independent experiments. The half maximal effective concentration (EC_{50}) values of BT-20, MDA-MB-468, and MDA-MB-231 cells treated with PEG engager^{CD19} plus Lipo-Vino or PEG engager^{EGFR} plus Lipo-Vino were analyzed (n = 3). Data are shown as mean ± standard deviation. Significant differences in mean EC_{50} values are indicated: *, $p \leq 0.01$, **, $p \leq 0.001$, ***, $p \leq 0.0001$ (two-way analysis of variance).

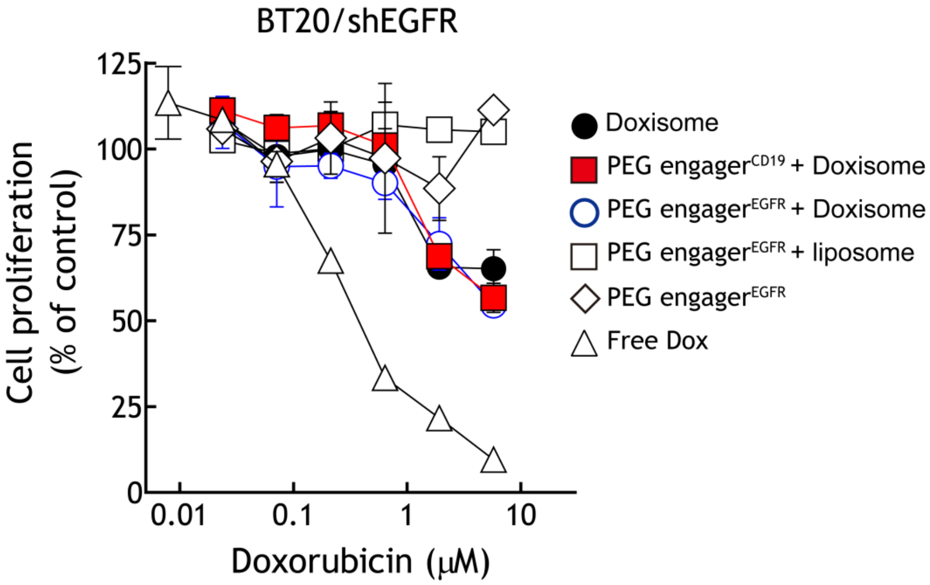

Supplementary Figure 6. PEG engager^{EGFR} mediated anti-tumor effects correlate with EGFR levels. BT20/shEGFR cells were incubated with PEG engager^{EGFR} (white circles), PEG engager^{CD19} (red squares), or culture medium (black circles) for 30 min before addition of serial dilutions of Doxisome® (liposomal doxorubicin). Serial dilutions of doxorubicin (white triangles), PEG engager^{EGFR} (white diamonds) or PEG engager^{EGFR} followed by serial dilutions of empty liposomes (white squares) were also added to cells in triplicate for 4 h. The incorporation of ^{3}H-thymidine into cellular DNA was measured 72 h later. The data are representative of three independent experiments. Data are shown as mean ± standard deviation.

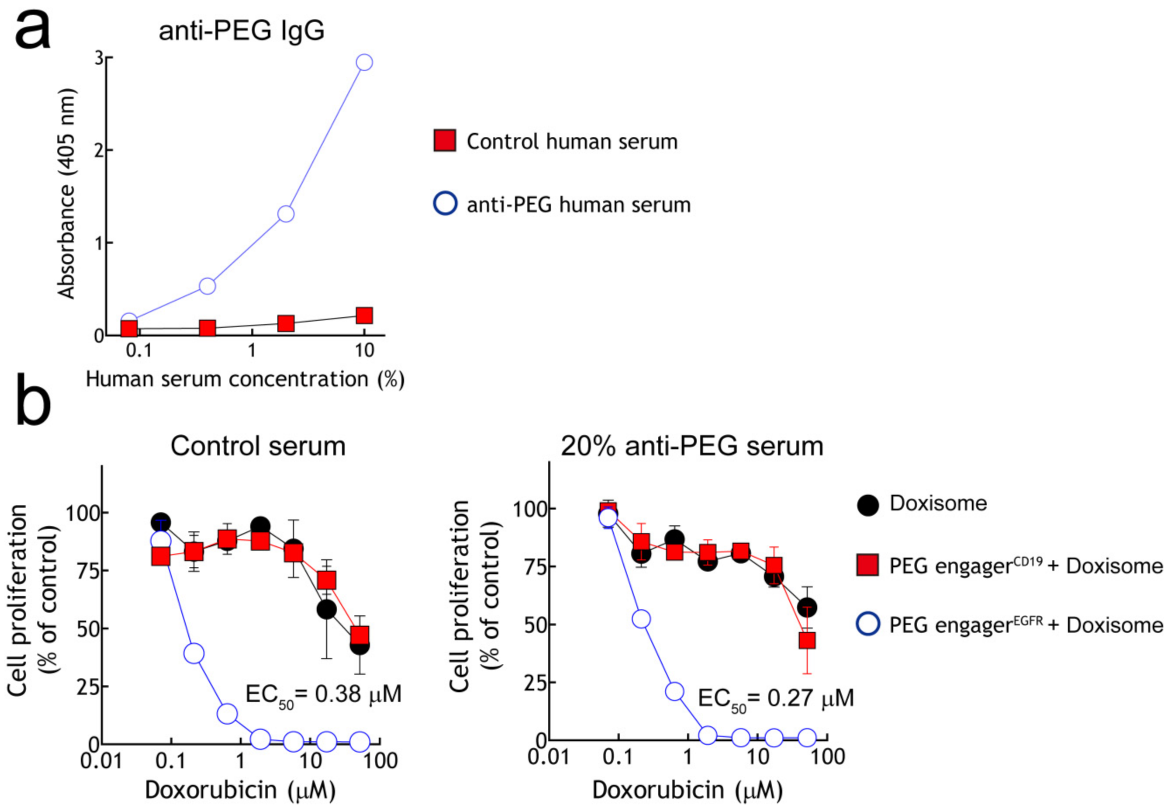

Supplementary Figure 7. Influence of anti-PEG antibodies on PEG engager. (a) Graded concentrations of the control human serum (red squares) or anti-PEG human serum (white circles) were incubated in PEG_{10K} coated microtiter plates. The plates were detected with anti-human IgG secondary antibodies. After washing, binding was determined by ELISA. Data are shown as mean ± standard deviation. (n = 3). (b) MDA-MB-468 cells were incubated with PEG engager^{EGFR} (white circles), PEG engager^{CD19} (red squares), or culture medium (black circles) for 30 min followed by serial dilutions of Doxisome® (liposomal doxorubicin) in the presence of 20% control serum or anti-PEG serum in triplicate for 4 h. The incorporation of ^{3}H-thymidine into cellular DNA was measured 72 h later (n = 3). The half maximal effective concentration (EC_{50}) values of MDA-MB-468 cells treated with PEG engager^{EGFR} plus Doxisome® were analyzed (n = 3). Data are shown as mean ± standard deviation.

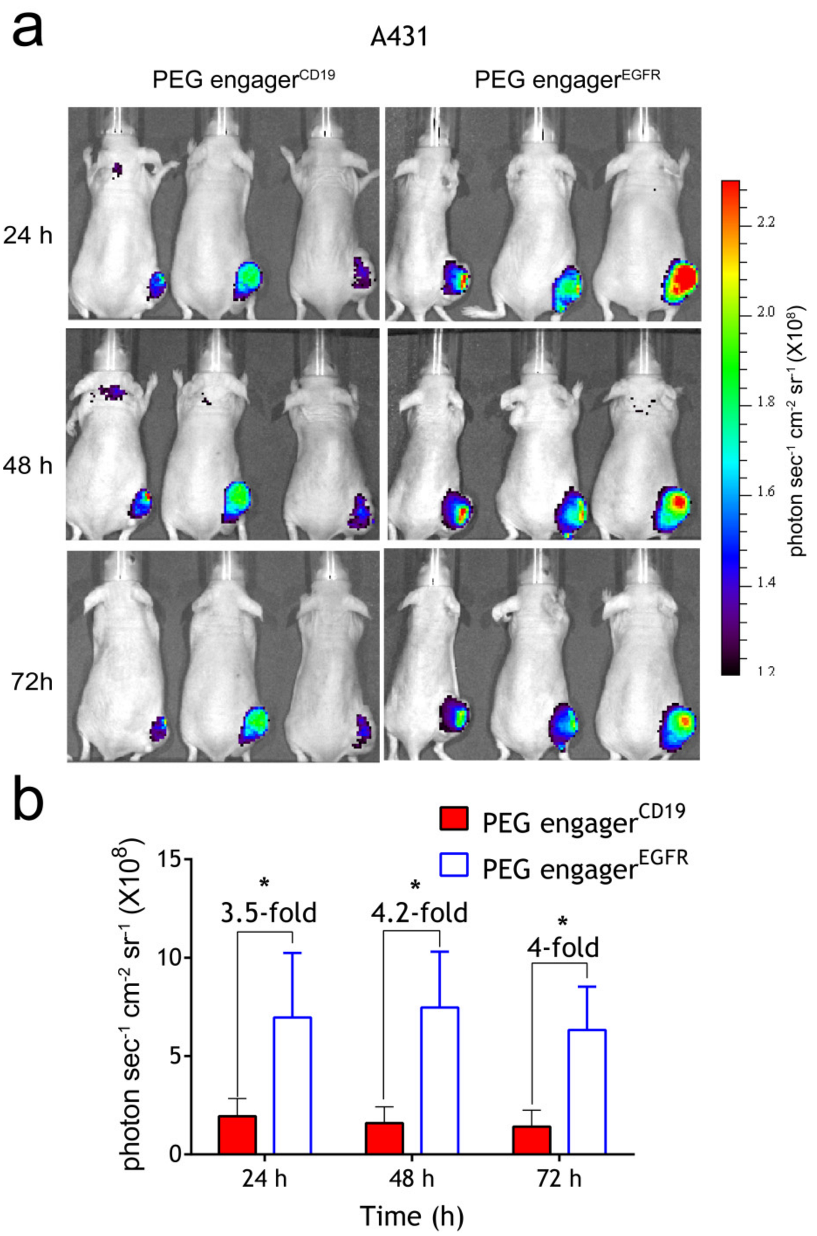

Supplementary Figure 8. Imaging of PEG engagers in A431 tumor-bearing mice. (a) Five hours before intravenous administration of 4armPEG$_{10k}$-NIR-797 probes (5 mg kg^{-1}), BALB/c nude mice bearing subcutaneous A431 tumors were intravenously injected with 6 mg kg^{-1} PEG engager^{EGFR} or PEG engager^{CD19} and the whole-body imaging were sequentially imaged at, 24, 48 and 72 h with an IVIS® spectrum imaging system. (b) The uptake of PEG-NIR797 in A431 tumors was determined by measuring fluorescence intensities (n = 3). Data are shown as mean ± standard deviation. Significant differences in mean fluorescent intensity between PEG engager^{EGFR} and PEG engager^{CD19} groups are indicated: *, $p \leq 0.01$ (two-way analysis of variance).

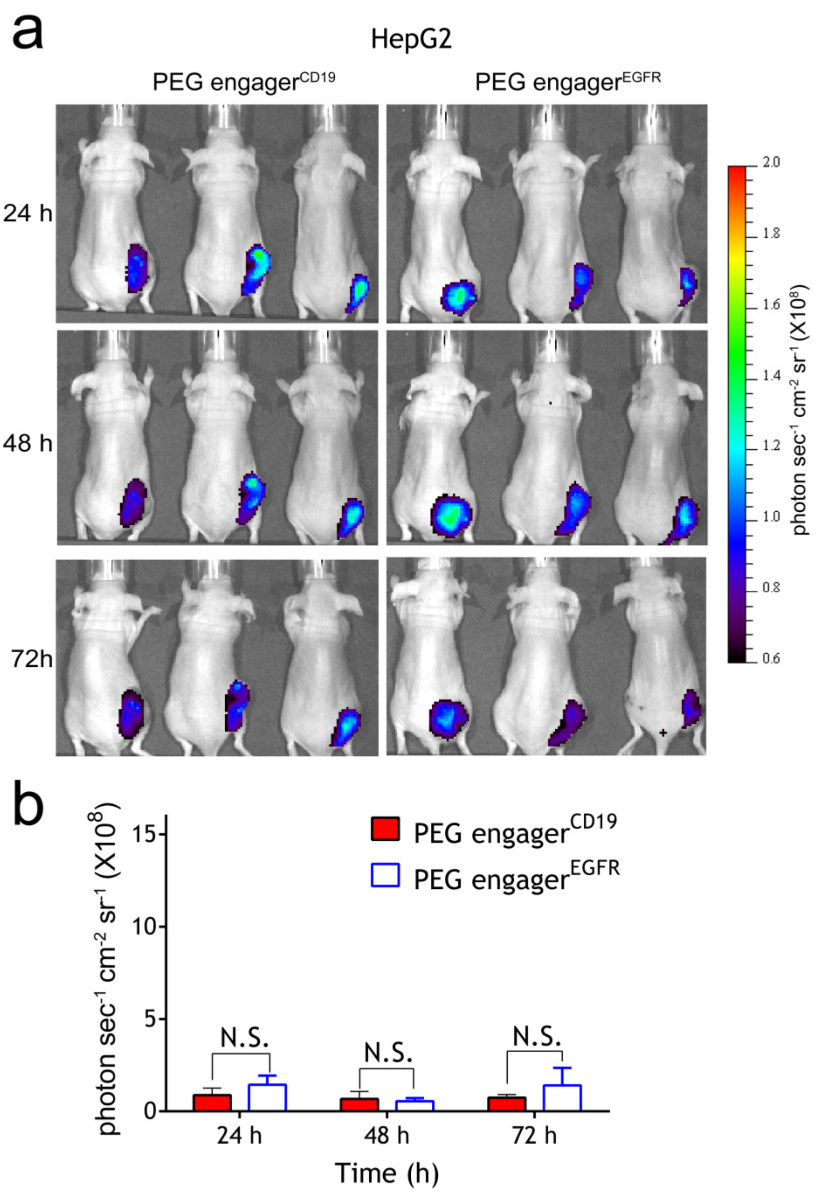

Supplementary Figure 9. Imaging of PEG engagers in HepG2 tumor-bearing mice. (a) Five hours before intravenous administration of 4armPEG$_{10k}$-NIR-797 probes (5 mg kg^{-1}), BALB/c nude mice bearing subcutaneous HepG2 tumors were intravenously injected with 6 mg kg^{-1} PEG engager^{EGFR} or PEG engager^{CD19} and the whole-body imaging were sequentially imaged at 24, 48 and 72 h with an IVIS® spectrum imaging system. (b) The uptake of PEG-NIR797 in HepG2 tumors was determined by measuring fluorescence intensities (n = 3). Data are shown as mean ± standard deviation. Significant differences in mean fluorescent intensity between PEG engager^{EGFR} and PEG engager^{CD19} groups are indicated: N.S., not significant (two-way analysis of variance).

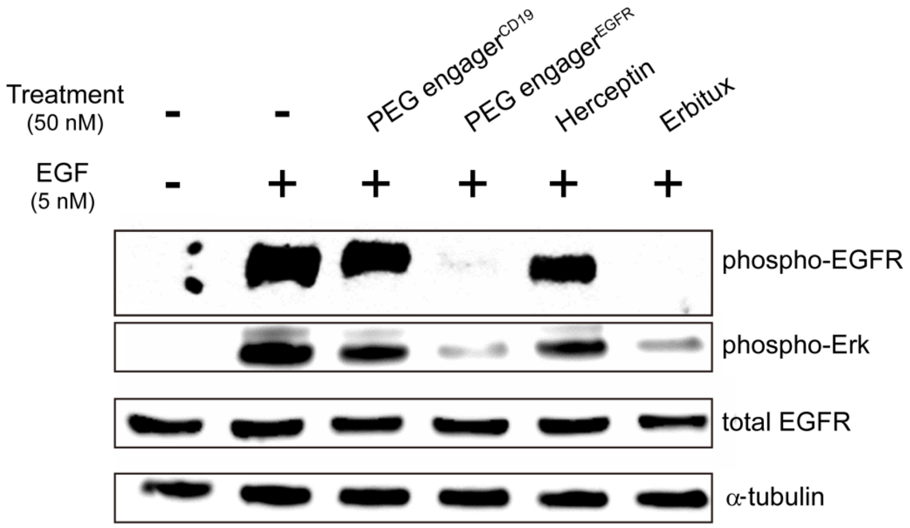

Supplementary Figure 10. PEG engager^{EGFR} blocks the EGFR signaling in EGFR-positive cells. Starved A431 cells (24 hours) were incubated with or without epidermal growth factor (EGF) and then sequentially treated with PEG engager^{CD19}, PEG engager^{EGFR}, Herceptin® (anti-HER2 antibody) or Erbitux® (anti-EGFR antibody). Phosphorylation of EGFR and Erk were detected by western blotting using anti-phospho EGFR or anti-phospho ERK antibodies. Total EGFR and tubulin served as loading controls.

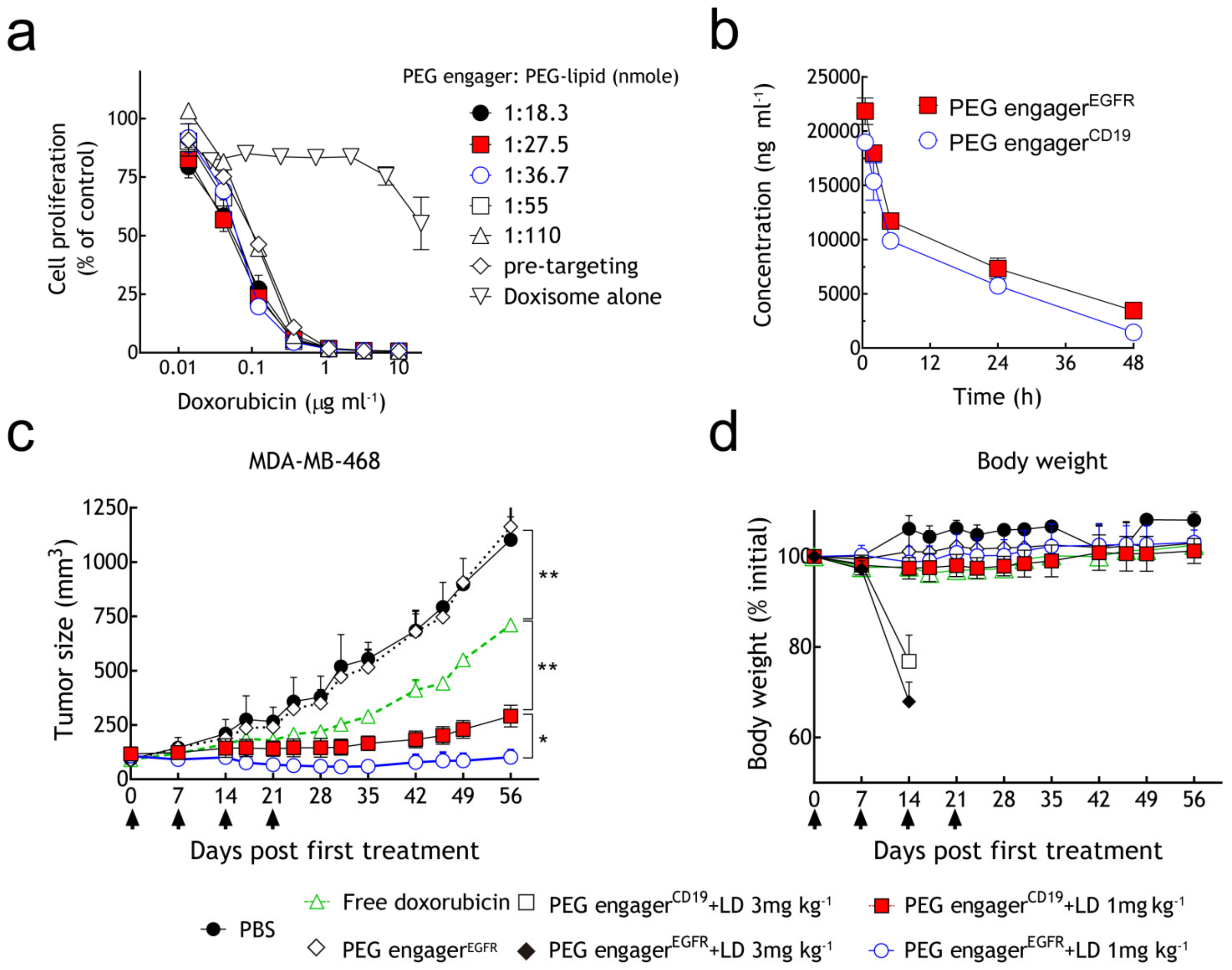

Supplementary Figure 11. Therapeutic efficiency of PEG engager modified Doxisome®. (a) PEG engager^{EGFR} and Doxisome® (liposomal doxorubicin) was premixed in different molar ratios of PEG engager and PEG-lipid on Doxisome® ranging from 1: 18.3 to 1: 110 at 4 ˚C for 1 hour. MDA-MB-468 cells were incubated with serial dilutions of Doxisome® alone (inverted triangles), pretargeted engager^{EGFR} followed by Doxisome® (white diamonds) or premixed PEG engager^{EGFR} /PEG-lipid on Doxisome® (1:18.3) (black circles), (1:27.5) (red squares), (1:37.6) (white circles), (1:55) (white squares), (1:110) (white triangles) in triplicate for 4 h. The incorporation of ^{3}H-thymidine into cellular DNA was measured 72 h later. (b) NOD SCID mice were intravenously injected with PEG engager decorated Doxisome® (3 mg kg^{-1}). Mean plasma concentrations of the PEG engagers were measured by sandwich ELISA (n = 3 mice). (c) PEG engagers were pre-mixed with Doxisome® at a molar ratio 1:55. Groups of eight NOD SCID mice bearing MDA-MB-468 tumors were intravenously injected with saline (black circles), 6 mg kg^{-1} PEG engager^{EGFR} (white diamonds), 1 mg kg^{-1} PEG engager^{EGFR}/Doxisome® (white circles), or 1 mg kg^{-1} PEG engager^{CD19}/Doxisome® (red squares), 3 mg kg^{-1} PEG engager^{EGFR}/Doxisome® (black diamonds) or 3 mg kg^{-1} PEG engager^{CD19}/Doxisome® (white squares) once a week for 4 weeks (arrows). Results show mean tumor sizes (n = 8). Data are shown as mean ± standard deviation. (d) Mean body weights of treated MDA-MB-468 mice (n = 8). Statistical analysis of the differences in tumor volumes between treatment and control groups was performed by one-way analysis of variance (ANOVA) followed by Dunnett's multiple comparisons. *, $p \leq 0.05$, **, $p \leq 0.005$.

Supplementary Methods

Size-exclusion high-performance liquid chromatography

Samples (50 µl, 2 mg ml^{-1}) were injected into an Aligent Bio SEC-5 column (300 × 7.8 mm, 300 Å) and separated at 1 ml per min in 50 mM sodium phosphate buffer, pH 7. Protein peaks were detected at 280 nm.

Thermal stability analysis of PEG engager antibodies

The PEG engager^{CD19} and PEG engager^{EGFR} in PBS were degassed and added into the sample chamber of a differential scanning calorimeter (Nano DSC III) (TA Instruments) at concentrations of 0.5 mg ml^{-1}. Degassed PBS was injected into the reference chamber. Differential power was monitored as each antibody-buffer pair was heated linearly from 10°C to 110°C at a rate of 1°C per minute under a fixed pressure of 3 atm. Buffer-buffer (degassed PBS) scans were also collected for baseline subtraction using the same procedure as for the antibody samples.

Short hairpin RNA transfection

The short hairpin RNA (shRNA) plasmid for the EGFR gene was obtained from the National RNAi Core Facility (Academia Sinica, Taipei, Taiwan). For EGFR knockdown, BT-20 cells were seeded overnight in 6-well plates at a density of 1 × 10^5 cells per well. Fresh medium without serum or antibiotics containing 5 µg ml^{-1} Polybrene (Sigma-Aldrich) and lentivirus carrying shRNA targeting EGFR (multiplicity of infection = 10, prepared by the National RNAi Corre Facility) was added to the cells for 24 h. After lentiviral infection, the cells were selected in 2 µg ml^{-1} puromycin for 4 days.

Human anti-PEG ELISA

Human serum samples containing pre-existing anti-PEG antibodies were screened from 1504 healthy donors using chimeric anti-PEG antibody reference standards developed in our lab[1]. Maxisorp 96-well microplates (Nalge-Nunc International, Rochester, NY) were coated with 0.5 µg per well NH_2-$PEG_{10,000}$-NH_2 in 50 µl per well 0.1 M $NaHCO_3$/Na_2CO_3 (adjusted to pH 9.5 with HCl) buffer overnight at 4°C and then blocked with 200 µl per well 5% skim milk in Dulbecco's phosphate-buffered saline (PBS, Thermo Fisher Scientific) at room temperature for 2 h. Plates were washed once with PBS immediately before use. Graded concentrations of human serum in 50 µl 2% skim milk in PBS was added to the NH_2-$PEG_{10,000}$-NH_2 (Sigma-Aldrich) coated plates at RT for 1 h. The plates were washed twice with 0.1 % CHAPS/PBS and once with PBS. 0.25 µg ml^{-1} horseradish peroxidase-conjugated goat $F(ab')_2$ anti-human IgG Fc (Jackson ImmunoResearch Laboratories) in 50 µl PBS containing 2% skim milk were added to the IgG plates, respectively for 1 h at room temperature. The plates were washed as above. Bound peroxidase activity was measured by adding 150 µl per well ABTS substrate solution (0.4 mg ml^{-1} 2,2′-azino-di(3-ethylbenzthiazoline-6-sulfonic acid), 0.003% H_2O_2, 100 mM phosphate citrate, pH 4.0) for 30 min at room temperature. The absorbance (405 nm) of wells was measured in a microplate reader (Molecular Device®).

Cell proliferation assay

Human serum containing a high titer of pre-existing anti-PEG IgG (relative concentration = 51.4 μg ml^{-1}) selected from 386 positive samples (mean concentration = 5.75 ± 16.0 μg ml^{-1}) was used to investigate whether pre-existing anti-PEG antibodies can influence the anti-proliferation efficacy of PEG engager-directed liposomal doxorubicin in TNBC cells. MDA-MB-468 cells (10,000 cells per well) were seeded in 96-well plates overnight. Fifteen microgram per ml of PEG $engager^{CD19}$ or PEG $engager^{EGFR}$ antibodies were added to the cells for 30 min at 37°C followed by addition of graded concentrations of PEGylated liposomal doxorubicin (Doxisome®, 13.9 μmol ml^{-1} lipid concentration, Taiwan Liposome Company Ltd., Taipei, Taiwan) to the cells in triplicate, with 20% control human serum or human serum containing pre-existing anti-PEG antibodies at 37°C for 4 h. The cells were subsequently washed once and incubated for an additional 72 h in fresh culture medium and then pulsed for 18 h with ^{3}H-thymidine (1 μCi per well). Results are expressed as percent inhibition of ^{3}H-thymidine incorporation into cellular DNA in comparison to untreated cells.

Western blot analysis

A431 cells were starved in DMEM without serum for 18 h. The cells (2×10^{5} cells per group) were detached with Accutase (Innovative Cell Technologies) and incubated with or without 50 nM of Herceptin® (anti-HER2, Genentech), Eributx® (anti-EGFR, Merck), PEG $engager^{CD19}$ or PEG $engager^{EGFR}$ prepared in PBS at 37 ˚C for 30 min prior to stimulation with or without recombinant human epidermal growth factor (5 nM, R&D Systems) at 37 ˚C for 5 min. The cells were lysed by using Pierce™ IP Lysis Buffer (ThermoFisher Scientific) containing Halt™ Protease and Phosphatase Inhibitor Cocktail (ThermoFisher Scientific). The protein concentration was analyzed using a BCA protein assay (ThermoFisher Scientific). Forty micrograms of total proteins were electrophoresed on a SDS-PAGE gel, transferred to a nitrocellulose membrane, and probed with anti-EGFR (Santa Cruz Biotechnology), anti-phospho EGFR (Tyr1068) (Cell Signaling Technology), anti-phospho Erk (Cell Signaling Technology), or anti-alpha tubulin (ThermoFisher Scientific) antibodies.

Optimized decoration of Doxisome® with PEG engagers

Doxisome® (Taiwan Liposome Company Ltd., Taipei, Taiwan) and PEG engagers were mixed at different molar ratio of protein to PEG-lipids ranging from 1: 18.3 to 1: 110 at 4 ˚C for 1 hour. MDA-MB-468 cells (10,000 cells per well) were seeded in 96-well plates overnight. Serial dilutions of PEG $engager^{EGFR}$ decorated Doxisome® was added to the cells in triplicate at 37°C for 4 h. The cells were subsequently washed once and incubated for an additional 72 h in fresh culture medium and then pulsed for 18 h with ^{3}H-thymidine (1 μCi per well). Results are expressed as percent inhibition of ^{3}H-thymidine incorporation into cellular DNA in comparison to untreated cells.

In vivo pharmacokinetics

NOD SCID mice were intravenously injected with PEG $engager^{CD19}$ or PEG $engager^{EGFR}$ decorated Doxisomes® (1.5 mg kg^{-1} of PEG engagers) and blood samples were periodically collected from the tail vein of the mice. Plasma was prepared by centrifugation (5 min, 12,000 × g). The PEG engager levels in

plasma were determined by quantitative sandwich ELISA. Maxisorp 96-well microplates were coated with 50 μl per well of anti-PEG antibody (AGP4)[2] (10 μg ml^{-1}) in bicarbonate buffer, pH 8.0 for 4 h at 37°C and then at 4°C overnight. The plates were blocked with 200 μl per well 5% skim milk in PBS for 2 h at room temperature and then washed with PBS three times. Serial dilutions of PEG engager decorated Doxisome® (as the standards) or plasma samples in dilution buffer (2% skim milk in PBS) were added to the wells for 2 h at room temperature. After washing with PBS four times, the plates were sequentially stained with 50 μl per well horseradish peroxidase-conjugated anti-human IgG Fab antibody (Jackson ImmunoResearch Laboratories) (5 μg ml^{-1}). The plates were washed with PBS six times and 100 μl per well ABTS solution (0.4 mg ml^{-1} 2,2´-azino-di(3-ethylbenzthiazoline-6-sulfonic acid), 0.003% H_2O_2, 100 mM phosphate citrate, pH 4.0) was added for 30 min at room temperature. The absorbance of the wells at 405 nm was measured on a microplate reader. The initial and terminal half-lives of the PEG engagers were estimated by fitting the data to a two-phase exponential decay model with Prism 5 software (Graphpad Software).

In vivo antitumor therapy with Doxisome® decorated with PEG engagers

Groups of NOD SCID mice (n= 8) bearing 84.3±4.3 mm^3 subcutaneous MDA-MB-468 tumors on their right flank were intravenously injected with PBS, PEG engager^{EGFR} alone (6 mg kg^{-1}), free doxorubicin (3 mg kg^{-1}), 3 mg kg^{-1} Doxisome® alone, PEG engager^{CD19} decorated Doxisome® (1 mg kg^{-1}) or PEG engager^{EGFR} decorated Doxisome® (1 mg kg^{-1}). Treatment was repeated once a week for a total of 4 weeks. Tumor sizes were measured every 7 days. Tumor volumes were calculated according the formula: length × width × height × 0.5.

Supplementary References

1. Chen, B. M., *et al.* Measurement of Pre-Existing IgG and IgM Antibodies against Polyethylene Glycol in Healthy Individuals. *Anal. Chem.* **88**, 10661-10666 (2016).

2. Su, Y. C., Chen, B. M., Chuang, K. H., Cheng, T. L., Roffler, S. R. Sensitive quantification of PEGylated compounds by second-generation anti-poly(ethylene glycol) monoclonal antibodies. *Bioconjug. Chem.* **21**, 1264-1270 (2010).
